# Predictors of Nonseroconversion after SARS-CoV-2 Infection

**DOI:** 10.3201/eid2802.211971

**Published:** 2022-02

**Authors:** Benjamin Davido, Karim Jaffal, Djillali Annane, Christine Lawrence, Elyanne Gault, Pierre De

**Affiliations:** Université Versailles-Saint-Quentin, Versailles, France (B. Davido, K. Jaffal, D. Annane);; Maladies infectieuses, Université Paris-Saclay, Garches, France (B. Davido, K. Jaffal, E. Gault, P. De Truchis);; Médecine Intensive et réanimation, Université Paris-Saclay, Boulogne-Billancourt, France (D. Annane);; Virologie, Université Paris-Saclay, AP-HP Hôpital Ambroise Paré, Boulogne-Billancourt (C. Lawrence, E. Gault)

**Keywords:** COVID-19, COVID vaccine, mRNA, reinfection, coronavirus diseases, coronavirus disease, SARS-CoV-2, severe acute respiratory syndrome coronavirus 2, respiratory infections, zoonoses, viruses

**To the Editor:** Recently, Liu et al. (*1*) described the predictors of nonseroconversion after severe acute respiratory syndrome coronavirus 2 (SARS-CoV-2) infection (36.1% of cases), where nonresponders had significant higher cycle threshold (C_t_) and were younger. Although a recent study showed that 1 dose of mRNA vaccine is sufficiently effective in previously infected persons (*2*), Reynolds et al. reported a previously infected vaccinee who never seroconverted (*3*). We report the case of a previously infected vaccinee who did not seroconvert and was subsequently reinfected.

In April 2020, a 55-year-old female nursing manager had mild SARS-CoV-2 pneumonia diagnosed that did not require admission, confirmed by weakly positive genes E and RNA-dependent RNA polymerase PCR testing (both C_t_ >33, near the limit of detection using homemade techniques). Concomitantly, her husband experienced symptoms and also tested positive, supporting that the woman’s case was not a false-positive. One month later, SARS-CoV-2 serology revealed no detectable antibodies to nucleocapsid or spike (S) proteins.

Despite a low risk for SARS-CoV-2 reinfection in a healthcare worker without underlying conditions (*4*) and having been vaccinated with 1 dose of mRNA BNT162b2 (Pfizer-BioNTech, https://www.pfizer.com) in April 2021, as recommended for previously infected persons, the woman was reinfected in September 2021 by the Delta variant. She had mild symptoms and a high estimated viral load (C_t_ 26 for genes E and N2). Serologic testing at the time of the first detection of reinfection revealed a relatively low titer of 20 binding antibody units/mL of S antibodies, which then increased to 243 BAU/mL 1 month after reinfection. Testing to rule out immune deficiency (serum protein electrophoresis, quantitative immunoglobulin assay, and assessment for complement deficiency) detected no abnormalities.

Our findings support a 2-dose vaccine policy for previously infected persons, as applied in the United States. This cautious approach is even more relevant because neutralizing antibody titers are substantially reduced in patients infected with the Delta variant (*5*) and in light of efforts to promote a third dose of vaccine, to ensure a stable antibody level over time in persons at high risk of being hospitalized for severe coronavirus disease.
